# Multilocus sequence typing identifies an avian-like *Chlamydia psittaci* strain involved in equine placentitis and associated with subsequent human psittacosis

**DOI:** 10.1038/emi.2016.135

**Published:** 2017-02-15

**Authors:** Martina Jelocnik, James Branley, Jane Heller, Shane Raidal, Susan Alderson, Francesca Galea, Melinda Gabor, Adam Polkinghorne

**Affiliations:** 1 Centre for Animal Health Innovation, Faculty of Science, Health, Education and Engineering, University of the Sunshine Coast, Sippy Downs, QLD 4556, Australia; 2 Nepean Hospital, Penrith, NSW 2751, Australia; 3 School of Animal and Veterinary Services, Faculty of Veterinary Sciences, Charles Sturt University, Wagga Wagga, NSW 2678, Australia; 4 Pathology West, Westmead Hospital, Westmead, NSW 2145, Australia; 5 State Veterinary Diagnostic Laboratory, NSW Department of Primary Industries, Menangle, NSW 2568, Australia

**Dear Editor,**

*Chlamydia psittaci* is an avian pathogen implicated in successful cross-host transmission to a range of hosts, causing a systemic disease known as psittacosis.^1^ In Australia and elsewhere, psittacosis is a notifiable disease.^2^ Molecular typing has suggested that Australian human *C. psittaci* isolates belong to a globally disseminated, highly virulent clonal 6BC clade,^3, 4^ primarily thought to be acquired through contact with *C. psittaci*-infected parrots.

In 2014, in regional New South Wales (NSW), Australia, nine staff and student veterinarians from a regional university and three staff from an equine stud farm were exposed to a set of abnormal equine fetal membranes, from which *C. psittaci* was identified. Following this exposure, a total of five cases of human psittacosis were subsequently diagnosed based on the presence of clinical signs of pneumonia, chest X-rays and/or serological testing (Chan J, 2016, unpubl. data). The genetic identity of the equine *C. psittaci* strain associated with these cases is unknown. In the present study, we report the isolation of *C. psittaci* from the placental material from this event and applied molecular typing in order to characterise this strain and to understand the epidemiology of this infection.

A placental sample was obtained from visually thickened abnormal placental membranes from a mare who delivered a live foal that died suddenly one week after being born ill on a Southern NSW farm, geographically isolated from locations of previously reported psittacosis. Microbiological diagnostic testing of the placental sample failed to detect other recognised pathogens. Histopathology revealed the presence of a mild, diffuse, interstitial placentitis and no evidence of an invasive bacterial infection. The sample was homogenised and *C. psittaci* was then isolated in Buffalo Green Monkey kidney cells, and passaged in the Physical Containment three laboratory at Pathology West, Westmead, NSW. The resulting isolate (Horse_pl) was heat-treated to render it non-infective prior to DNA extraction using a Becton Dickinson MAX extraction (Becton Dickinson, North Ryde, Sydney, Australia), following a *C. psittaci*-specific quantitative PCR targeting the *incA* gene to confirm the identity of the isolate.^2, 5^ A conventional PCR to generate a 298-base-pair (bp) amplicon of the *Chlamydiales* signature 16S ribosomal DNA sequence was also performed^6^ to independently validate the species-specific quantitative PCR. Positive (previously described avian *C. psittaci* CROO9 DNA) and negative (dH_2_O) controls were included in each assay.

A *C. psittaci*-specific multilocus sequence typing (MLST) scheme, targeting seven house-keeping gene fragments,^7^ was applied to the placental isolate and a parrot CR394 strain available in our collection. We also generated the complete gene sequence of the chlamydial major outer membrane gene (*omp*A) for the Horse_pl isolate using previously described *C. psittaci*-specific primers, CTU and ompA-rev.^8^ All amplicons were purified using a Roche High Pure kit (Roche, Sydney, NSW, Australia) and sent for Sanger sequencing (Macrogen, Korea).

Sequence analyses were performed in Geneious R9.^9^ The Horse_pl *omp*A sequence was analysed by BLASTn, and aligned using ClustalW (as implemented in Geneious) to other publically available *C. psittaci omp*A sequences retrieved from GenBank (https://www.ncbi.nlm.nih.gov/genbank/). The sequence types for the *C. psittaci* Horse_pl and CR394 described in this study were determined from the *Chlamydiales* MLST database (http://pubmlst.org/chlamydiales/). MLST sequences from an additional 25 *C. psittaci* strains were also available from the *Chlamydiales* MLST database. A Bayesian phylogenetic tree constructed from the alignment of the concatenated MLST sequences for the 26 *C. psittaci* isolates used in our analyses was constructed with MrBayes.^10^ Tree parameters included GTR+G nucleotide substitution model, with four Markov Chain Monte Carlo chains of million generations, subsampled every 1000 runs, and 10 000 trees discarded. CR394 MLST and a partial 16S rDNA sequence, and Horse_pl MLST, *omp*A and a partial 16S rDNA sequences were deposited in Genbank (Accession numbers KX834217—KX834231, and KY287780—KY287781).

Nucleotide BLAST analyses of the 298 bp 16S rDNA sequence amplified from the *C. psittaci* Horse_pl genomic DNA extracts revealed 100% sequence identity to various *C. psittaci* 16S rRNA gene sequences, confirming that the chlamydial strain isolated was indeed *C. psittaci*. Subsequent *C. psittaci* MLST typing and phylogenetic analyses revealed that the *C. psittaci* Horse_pl isolate is a *C. psittaci* ST 24 type strain, belonging to the highly virulent *C. psittaci* 6BC lineage^3, 4^ (Figure 1). The Horse_pl MLST sequence was identical to the 99DC5 strain, previously isolated from a mare from Germany, as well as to those from recently described Australian parrot and human *C. psittaci* strains^4^ and the additional Australian parrot CR394 strain available in our collection. These Australian strains were isolated from an endemic area in NSW, where a cluster of human cases presented with serious illness and had direct as well as indirect contact with parrots. Congruent with these observation, the *C. psittaci* Horse_pl *omp*A sequence was found to be identical to the *omp*A sequences from previously described Australian isolates, but also to other avian, livestock and human isolates denoted *C. psittaci* Genotype A, subgroup 84/55, based on a typing system proposed by Sachse *et al.*^8^ The Horse_pl *omp*A sequence shared 99.8% and 99.0% identity to the *omp*A sequences from the remaining subgroups 6BC and MN Zhang within Genotype A, respectively.^8^ In comparison with the *omp*A sequence from the German equine 99DC5 strain, the Horse_pl *omp*A sequence differed by two nucleotides (99.8% sequence identity). Interestingly, based on the previously proposed *C. psittaci omp*A genotyping scheme,^8^ equine *C. psittaci* 99DC5 *omp*A represents a novel subgroup within Genotype A, differing by two nucleotides from subgroups 6BC and 84/55, and 11 nucleotides from subgroup MN.

The molecular description of a *C. psittaci* strain implicated in abnormal equine fetal membranes in association with neonatal mortality raises serious questions about the impact of this pathogen in horses but also the public health risk of human exposure. The revelation that this isolate belongs to the highly virulent 6BC clade of *C. psittaci* strongly suggests that its original reservoir may have been a native Australian psittacine. On the basis of our recent molecular typing studies of human and parrot *C. psittaci* strains,^4^ we have previously postulated that *C. psittaci* 6BC strains can be also transmitted via indirect contact, presumably via faecal environmental contamination from *C. psittaci*-infected parrots.

The potential role of *C. psittaci* in equine abortions has been previously recognised in Europe,^11^ although the identity of infecting strains was unknown. In the Australian context, whether this is an isolated case or may be more common is unclear—certainly, *C. psittaci* infection is not routinely considered in the differential diagnosis of equine abortion. Of more significant concern is the association between contact with *C. psittaci* 6BC-type-infected placental material and the suspected zoonotic transmission to humans in this case (Chan J, 2016, unpubl. data). *C. psittaci* 6BC strains have been previously shown to readily infect and cause serious human disease;^3, 4, 12^ however, this appears to be the first report suggesting that contact with infected horses and/or equine products may be a source of transmission of this pathogen.

This work highlights the additional public health precautions that veterinarians and others coming into contact with suspected *C. psittaci*-infected equine products of conceptus must employ to reduce the potential risk of zoonoses. More investigations to confirm the zoonotic character of the human infection and confirm the potential reservoirs of equine *C. psittaci* infection will be required to reduce the risk of these infections to horses and humans.

## Figures and Tables

**Figure 1:**
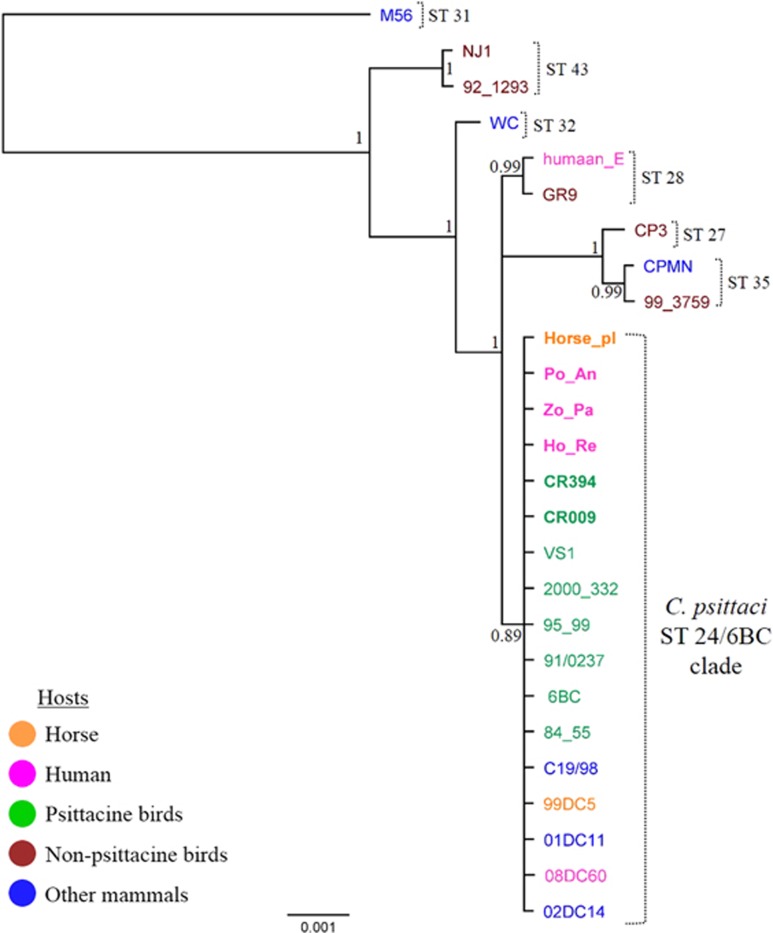
Mid-point rooted Bayesian phylogenetic analysis of the concatenated seven MLST gene fragment sequences from 26 *C. psittaci* strains from various hosts, including *C. psittaci* Horse_pl strain described here. Australian *C. psittaci* isolates are denoted in bold. Hosts are indicated by the colours in the legend. Posterior probabilities are displayed on the tree nodes. MLST, multilocus sequence typing.
